# Vaping-associated esophagitis

**DOI:** 10.1186/s12876-021-01695-8

**Published:** 2021-03-05

**Authors:** Trisha Satya Pasricha, Bharati Kochar

**Affiliations:** 1 Division of Gastroenterology, Massachusetts General Hospital and Harvard Medical School Boston, 55 Fruit Street, Boston, MA 02114 USA; 2 Clinical Translational Epidemiology Unit, The Mongan Institute, Boston, MA USA

**Keywords:** Vaping, Esophagitis, Odynophagia, E-cigarettes, Case report

## Abstract

**Background:**

Vaping, or e-cigarettes, heat nicotine and other chemicals to create a vapor that is inhaled. The practice has gained rapid popularity with 41 million people globally reporting regular or occasional use. Although tobacco smoking is well-known to increase esophageal acid exposure by augmenting the number of reflux events, the effects of vaping on the gastrointestinal tract have not yet been elucidated. Our objective is to report a case of severe esophagitis associated with vaping, which is the first in the literature to our knowledge.

**Case presentation:**

A 25-year-old male with a history of well-controlled gastro-esophageal reflux disease presented to the emergency room for evaluation of one week of severe odynophagia. He had been treated with a proton-pump inhibitor for several years with good effect. Approximately two months prior to presentation, he started vaping tetrahydrocannabinol and nicotine with recent heavy daily use. He denied any alcohol or non-steroidal anti-inflammatory drug use. We performed esophagogastroduodenoscopy that revealed Los Angeles Grade C esophagitis (involving ≥ 1 mucosal breaks continuous between tops of ≥ 2 mucosal folds, < 75% circumferential). Histopathological analysis of esophageal biopsies demonstrated granulation tissue with acute and chronic inflammation. Periodic acid-Schiff-diastase staining was negative and immunohistochemical stains for herpes simplex virus and cytomegalovirus were negative. There was no evidence of eosinophilic esophagitis. We treated him with intravenous PPI and analgesics until he was able to tolerate oral intake. He was counseled extensively on vaping cessation and reported complete resolution of symptoms after 2 months.

**Conclusion:**

This patient’s presentation illustrates a serious gastrointestinal consequence of vaping, the long-term consequences of which warrant additional studies. Like smoking, the mechanism of injury in vaping may be, at least in part, due to the effects of nicotine. As prevalence of vaping continues to rise, clinicians should be aware of this complication and carefully solicit a patient’s vaping history as a simple denial of “smoking” can be misleading.

## Background

Vaping, or e-cigarettes, heat nicotine and other chemicals to create a vapor that is inhaled. The practice has gained rapid popularity with 41 million people globally reporting regular or occasional use [1]. Although tobacco smoking is well-known to increase esophageal acid exposure by augmenting the number of reflux events [5], the effects of vaping on the gastrointestinal tract have not yet been elucidated. Our objectives are to report a case of severe esophagitis associated with vaping, which is the first in the literature to our knowledge.

## Case presentation

A 25-year-old male with a history of well-controlled gastro-esophageal reflux disease (GERD) presented to the emergency room for evaluation of one week of severe odynophagia and inability to tolerate po. He had been treated with a proton-pump inhibitor (PPI) for several years with good effect. Approximately 2 months prior to presentation, he started vaping tetrahydrocannabinol (THC) and nicotine with recent heavy daily use. He denied any alcohol or NSAID intake. On physical exam, the patient was non-toxic appearing with a soft abdomen.

**Fig. 1:**
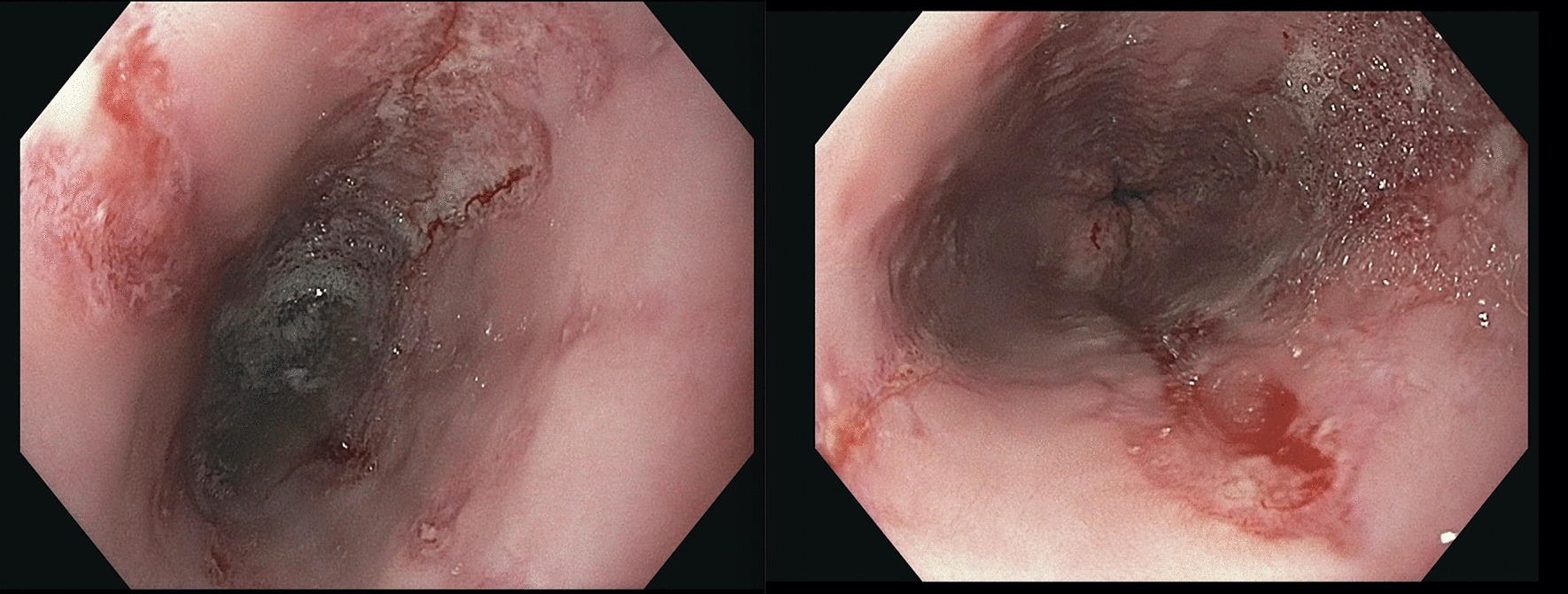
Images of the esophagus during esophagogastroduodenoscopy. Images of the patient’s esophagus obtained during esophagogastroduodenoscopy demonstrating severe bridging mucosal breaks less than 75% of the circumference, consistent with Grade C esophagitis per the Los Angeles Criteria for erosive esophagitis

**Fig. 2:**
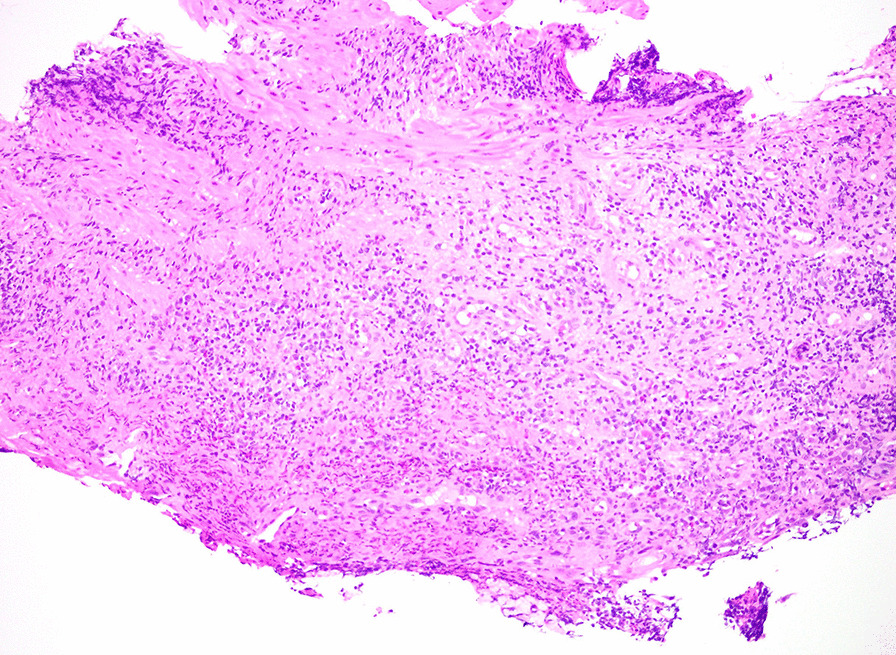
Histological image of the distal esophageal biopsy specimen. Hematoxylin and eosin stain of the patient’s distal esophageal mucosal biopsy specimen demonstrating granulation tissue with acute on chronic inflammation. Photograph provided by Dr. Vikram Deshpande (Massachusetts General Hospital)

We performed esophagogastroduodenoscopy that revealed Los Angeles Grade C esophagitis (involving ≥ 1 mucosal breaks continuous between tops of ≥ 2 mucosal folds, < 75% circumferential) (Fig. 1). Histopathological analysis of esophageal biopsies demonstrated granulation tissue with acute and chronic inflammation (Fig. 2). Periodic acid-Schiff-diastase staining was negative and immunohistochemical stains for herpes simplex virus and cytomegalovirus were negative. There was no evidence of eosinophilic esophagitis. He was diagnosed with esophagitis secondary to vaping. We treated him with intravenous 40 mg twice daily PPI and analgesics until he was able to tolerate oral intake. He was counseled extensively on vaping cessation. The patient reported complete resolution of symptoms after 2 months of PPI therapy and vaping cessation.

## Discussion and conclusion

The emerging consequences of vaping have garnered significant public health attention in the product’s largest markets, the United States, the United Kingdom and France, which spend a combined $10 billion yearly on vaping products [1]. In addition to well-publicized lung injury, vaping is also associated with increased cardiovascular disease [2] and the development of oral ulceration [3].

The temporality of his commencing vaping with his symptoms strongly suggested a relationship, supported by the endoscopic appearance, histopathologic analysis, and exclusion of other etiologies. Cigarette smoking classically increases the risk of Barrett’s esophagus and malignancy, which it does synergistically with GERD [4]. Indeed cigarette smoking increases the odds of gastro-esophageal reflux symptoms [5] and greatly increases acid exposure time on ambulatory pH monitoring [6]—effects which are largely attributed to nicotine. Similarly, the mechanism of injury in vaping may be, at least in part, due to the effects of nicotine when present in the vaping substrate. Interestingly, like nicotine [7], THC (and namely, activation of cannabinoid receptors 1 and 2) has also been shown to play in important role in the regulation of transient lower esophageal sphincter relaxations, as well as lower esophageal pressure [8, 9], further supporting vaping as the trigger of our patient’s presentation. Vaping poses particular challenges given the risk of unknown chemicals and toxins entering the substrate due to inconsistent regulations. However, independent of the substrate itself, direct mucosal injury as another mechanism of injury has been proposed secondary to the by-products of vaporized additives, which result in oxidative stress and DNA damage [10].

This patient’s presentation illustrates a serious gastrointestinal consequence of vaping, the long-term consequences of which warrant additional studies. As prevalence of vaping continues to rise, clinicians should be aware of this complication and carefully solicit a patient’s vaping history as a simple denial of “smoking” can be misleading.

## Data Availability

Data sharing not applicable to this article as no datasets were generated or analysed during the current study.

## Abbreviations

GERD: Gastroesophageal reflux disease
PPI: Proton-pump inhibitor
THC: Tetrahydrocannabinol
NSAID: Non-steroidal anti-inflammatory drug

## References

[1] Jones L. Vaping: how popular are e-cigarettes? *BBC News*, BBC, 14 Sept. 2019, www.bbc.com/news/business-44295336.

[2] Osei AD, Mirbolouk M, Orimoloye OA, Dzaye O, Uddin SMI, Benjamin EJ, Hall ME, DeFilippis AP, Stokes A, Bhatnagar A, Nasir K, Blaha MJ (2019). Association between e-cigarette use and cardiovascular disease among never and current combustible-cigarette smokers. Am J Med.

[3] Ali NS, Billings ML, Tollefson MM, Davis DMR, Hand JL (2020). Oral erosions associated with surreptitious marijuana vaping in an adolescent boy. Pediatr Dermatol.

[4] Cook MB, Shaheen NJ, Anderson LA, Giffen C, Chow WH, Vaughan TL, Whiteman DC, Corley DA (2012). Cigarette smoking increases risk of Barrett's esophagus: an analysis of the Barrett's and Esophageal Adenocarcinoma Consortium. Gastroenterology.

[5] Eusebi LH, Ratnakumaran R, Yuan Y, Solaymani-Dodaran M, Bazzoli F, Ford AC (2018). Global prevalence of, and risk factors for, gastro-oesophageal reflux symptoms: a meta-analysis. Gut.

[6] Kadakia SC, Kikendall JW, Maydonovitch C, Johnson LF (1995). Effect of cigarette smoking on gastroesophageal reflux measured by 24-h ambulatory esophageal pH monitoring. Am J Gastroenterol.

[7] Kahrilas PJ, Gupta RR (1990). Mechanisms of acid reflux associated with cigarette smoking. Gut.

[8] Beaumont H, Jensen J, Carlsson A, Ruth M, Lehmann A, Boeckxstaens G (2009). Effect of delta9-tetrahydrocannabinol, a cannabinoid receptor agonist, on the triggering of transient lower oesophageal sphincter relaxations in dogs and humans. Br J Pharmacol.

[9] Izzo AA, Camilleri M (2008). Emerging role of cannabinoids in gastrointestinal and liver diseases: basic and clinical aspects. Gut.

[10] Sundar IK, Javed F, Romanos GE, Rahman I (2016). E-cigarettes and flavorings induce inflammatory and pro-senescence responses in oral epithelial cells and periodontal fibroblasts. Oncotarget.

